# Consortia-mediated bioprocessing of cellulose to ethanol with a symbiotic *Clostridium phytofermentans*/yeast co-culture

**DOI:** 10.1186/1754-6834-6-59

**Published:** 2013-04-29

**Authors:** Trevor R Zuroff, Salvador Barri Xiques, Wayne R Curtis

**Affiliations:** 1 Current address: The Pennsylvania State University, 158 Fenske Laboratory, University Park, PA, 16802, USA; 2 Industrial Engineering Department, ETS IQS, Via Augusta 390, Barcelona, 08017, Spain

**Keywords:** Consortia, Consolidated bioprocessing, Cellulosic ethanol, Symbiosis, Oxygen transport

## Abstract

**Background:**

Lignocellulosic ethanol is a viable alternative to petroleum-based fuels with the added benefit of potentially lower greenhouse gas emissions. Consolidated bioprocessing (simultaneous enzyme production, hydrolysis and fermentation; CBP) is thought to be a low-cost processing scheme for lignocellulosic ethanol production. However, no single organism has been developed which is capable of high productivity, yield and titer ethanol production directly from lignocellulose. Consortia of cellulolytic and ethanologenic organisms could be an attractive alternate to the typical single organism approaches but implementation of consortia has a number of challenges (e.g., control, stability, productivity).

**Results:**

Ethanol is produced from α-cellulose using a consortium of *C. phytofermentans* and yeast that is maintained by controlled oxygen transport. Both *Saccharomyces cerevisiae* cdt-1 and *Candida molischiana* “protect” *C. phytofermentans* from introduced oxygen in return for soluble sugars released by *C. phytofermentans* hydrolysis. Only co-cultures were able to degrade filter paper when mono- and co-cultures were incubated at 30°C under semi-aerobic conditions. Using controlled oxygen delivery by diffusion through neoprene tubing at a calculated rate of approximately 8 μmol/L hour, we demonstrate establishment of the symbiotic relationship between *C. phytofermentans* and *S. cerevisiae* cdt-1 and maintenance of populations of 10^5^ to 10^6^ CFU/mL for 50 days. Comparable symbiotic population dynamics were observed in scaled up 500 mL bioreactors as those in 50 mL shake cultures. The conversion of α-cellulose to ethanol was shown to improve with additional cellulase indicating a limitation in hydrolysis rate. A co-culture of *C. phytofermentans* and *S. cerevisiae* cdt-1 with added endoglucanase produced approximately 22 g/L ethanol from 100 g/L α-cellulose compared to *C. phytofermentans* and *S. cerevisiae* cdt-1 mono-cultures which produced approximately 6 and 9 g/L, respectively.

**Conclusion:**

This work represents a significant step toward developing consortia-based bioprocessing systems for lignocellulosic biofuels production which utilize scalable, environmentally-mediated symbiosis mechanisms to provide consortium stability.

## Background

In nature microbes rarely live in isolation, but rather exist in highly diverse and complex communities referred to as consortia [1]. These consortia are often capable of tasks that are far too complex for any single organism to complete themselves including some of the most important global biogeochemical cycles [2]. The organisms living in these communities interact in numerous ways ranging from cooperation to direct competition [3]. Microbiologists and engineers have come to appreciate the diversity and capacity of natural microbial communities and large efforts have been undertaken to understand natural consortia and to engineer synthetic consortia for biotechnological purposes [4-7].

Two of the key challenges in society today are to reduce energy dependence on petroleum and reduce greenhouse gas emissions [8]. The transportation sector is a prime candidate for addressing these two challenges because it relies on petroleum for approximately 93% of its energy and releases almost as much carbon dioxide as both the commercial and residential sectors combined [9]. Lignocellulosic biomass has become an increasingly feasible source of carbohydrates for biological production of alternative fuels such as ethanol [10]. Consolidated bioprocessing (the simultaneous biological hydrolysis and fermentation of biomass; CBP) is thought to be one of the most cost effective means of producing ethanol from lignocellulose [11,12]. However, no single organism has been isolated or genetically engineered to reach high enough ethanol concentrations, yields and productivities from lignocellulose [13]. Natural microbial consortia, on the other hand, are innately capable of high conversion of lignocellulosic biomass [see Table one in reference [7] but the resultant products (e.g., organic acids, CO_2_, CH_4_) are not suitable large-scale liquid transportation fuels.

An elegant example of a naturally occurring, lignocellulose degrading microbial consortium is the symbionts of the termite hindgut. Complex macromolecules are deconstructed in a series of steps that are facilitated by specific microbial species [14]. Protists produce cellulases for cellulose hydrolysis, while nitrogen fixing bacteria (e.g., Clostridia) sequester atmospheric nitrogen to compensate for the low nitrogen content of wood. Fermentative organisms consume soluble sugars released from cellulose hydrolysis to produce organic acids, CO_2_ and H_2_ which are subsequently converted by methanogens to methane [15]. This, along with other examples of natural microbial communities, has prompted interest in utilization of microbial consortia for lignocellulosic biofuels production [5,7]. However, unlike these natural systems, scientists are currently limited in our ability to generate stable, productive microbial communities. In order to successfully implement large scale consolidated bioprocessing of lignocellulosic materials for fuel ethanol production we must develop stable microbial consortia with the necessary functionality, process control and efficiency.

To transition from natural consortia which contain potentially hundreds of organisms to synthetic consortia containing several defined species, one must develop a mechanism for population control. Generating stable, controllable consortia has been a focus of recent work to genetically engineer mechanisms for establishing intra-species consortia [16-19]. These approaches range from growth-controlling genetic circuits based on quorum sensing compounds [19] to complimentary auxotrophic amino acid exchange [17]. As proof-of-concept, these studies are quite elegant and encouraging but they may suffer on the large scale due to unstable and difficult genetic modifications in industrially relevant organisms. Others have demonstrated syntrophic interactions in a co-culture of *Actinotalea fermentans* with an engineered *S. cerevisiae* which produces methyl halides directly from cellulose [20]. *S. cerevisiae* relieves acetate inhibition by converting it to methyl halides, which are precursors for various fuel compounds. However, synthetic syntrophic interactions may be unstable since at least one organism does not necessarily rely on the other for survival. Without an additional level of control (e.g., spatial structure [21-23]) the community may breakdown. Obligate mutualisms such as those described by You et al. 2004 and Shou et al. 2007 may be a more stable approach for consortia-mediated lignocellulosic ethanol production. With this in mind, symbiotic consortia of a wide range of organisms could be generated using various mechanisms based on both genetic and environmental control factors [7].

In this work we develop a symbiotic co-culture of the cellulolytic mesophile, *Clostridium phytofermentans*[24] and a cellodextrin fermenting yeast, *Candida molischiana*[25] or *S. cerevisiae* cdt-1 [26]. We establish a symbiosis (obligate mutualism) between *C. phytofermentans* and the yeast species by controlling the volumetric transport rate of oxygen. Both yeasts are capable of providing respiratory protection to the obligate anaerobe, *C. phytofermentans*, in return for soluble carbohydrates released from cellulose hydrolysis. The yeast converts these soluble carbohydrates to ethanol. At high substrate loading we noted a decreased conversion of cellulose by *C. phytofermentans* therefore endoglucanase was added to further evaluate the potential for improvements in the co-culture approach. In this cellulase-assisted format, the co-culture produces over twice as much ethanol and degrades two and three times as much α-cellulose as the *S. cerevisiae* cdt-1 and *C. phytofermentans* mono-cultures, respectively. This work represents a significant step in utilizing a scalable environmental control mechanism (i.e. oxygen transport) to induce a stable symbiosis in a consortium of two diverse organisms. This general approach to symbiosis development is applicable to a variety of organisms, substrates and products and can be used to explore diverse consortia-mediated bioprocesses.

## Results and discussion

### Design and development of *C. phytofermentans*/yeast symbiosis

Oxygen introduction was proposed as a mechanism for establishing a symbiosis between mixtures of organisms in which one organism “protects” the other from oxygen inhibition in return for soluble carbohydrates released from cellulose [7]. In this work we investigated this mechanism and the subsequent performance of a combination of the cellulolytic obligate anaerobic mesophile, *C. phytofermentans*, with one of two cellodextrin fermenting yeasts, *C. molischiana* or *S. cerevisiae* cdt-1. *C. phytofermentans* was chosen for its ability to hydrolyze and ferment pure cellulose and natural lignocellulosic materials. For example, under consolidated bioprocessing (CBP) conditions (i.e., simultaneous biological hydrolysis and fermentation), *C. phytofermentans* was shown to hydrolyze 76% of glucan and 88.6% of xylan in AFEX treated corn stover and ferment it to 2.8 g/L ethanol in 10 days [27]. *C. molischiana* is naturally able to ferment cellodextrins with degree of polymerization 2 to 6 to ethanol with yields of approximately 0.40 g ethanol/g substrate in 44 hours [28]. *S. cerevisiae* cdt-1 was engineered to import and cleave cellodextrins for subsequent fermentation (yield of approximately 0.44 g/g cellobiose in about 100 hours) through expression of cellodextrin transporters and β-glucosidase from *Neurospora crassa*[26]. Although similar, the yeasts do have some important differences. For example, *S. cerevisiae* cdt-1 transports and cleaves cellodextrins internally [26] while *C. molischiana* does so externally with extracytoplasmic β-glucosidase [29]. These yeasts were chosen for their unique ability to ferment cellodextrins (not only glucose) which was hypothesized to increase ethanol yield and relieve cellulase inhibition by the soluble sugar products. In addition, these two organisms provide a comparison across two genus and between engineered and naturally occurring strains. In this study we used α-cellulose (Sigma) as a model cellulosic substrate to study hydrolysis and fermentation since it avoids the presence of additional carbon sources (e.g., hemicellulose).

**Figure 1:**
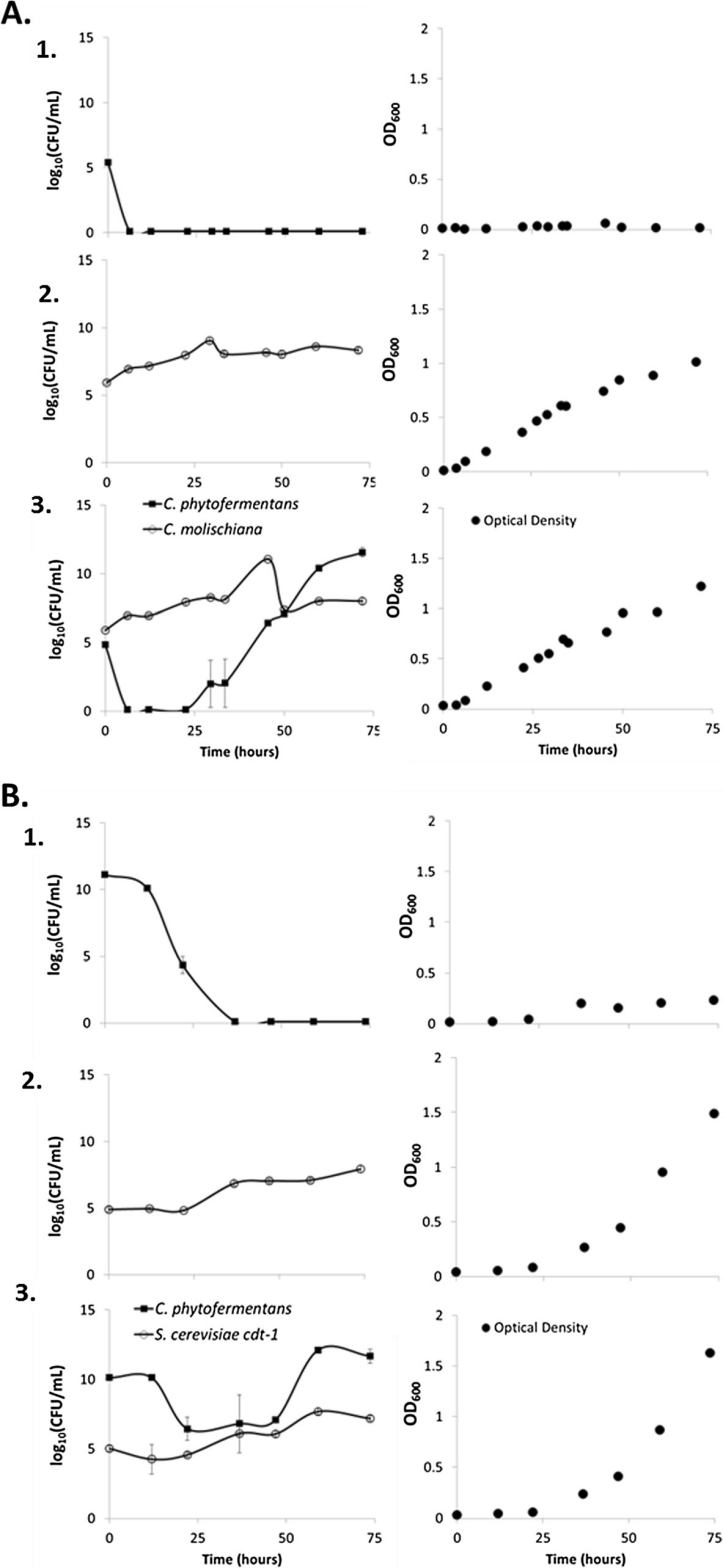
**Semi-aerobic mono- and co-culture growth on cellobiose.***C. phytofermentans* mono-culture **(A.1** and **B.1)**, *C. molischiana* mono-culture **(A.2)**,*C. phytofermentans*/*C. molischiana* co-culture **(A.3)**, *S. cerevisiae* cdt-1 mono-culture **(B.2)** and *C. phytofermentans*/*S. cerevisiae* cdt-1 co-culture **(B.3)** viable cell counts (left) and optical densities (right) in GS2 cellobiose fermentations. Results are representative of at least two independent experiments. Error bars are the standard deviation among drops for colony counting.

To test oxygen consumption and the resultant protection, cultures with 12 g/L cellobiose as a mutually accessible carbon source, were inoculated with 10^5^ to 10^6^ CFU/mL of *C. molischiana* or *S. cerevisiae* cdt-1 and 10^5^ to 10^10^ CFU/mL *C. phytofermentans*. Dissolved oxygen was not removed from the medium at the beginning of the experiment however the cap was kept tight throughout the experiment aside from sampling. *C. phytofermentans* mono-cultures were unable to grow and viable cells dropped below detection (< 100 CFU/mL) almost immediately with low starting concentration (Figure 1A.1.) and after a short lag with higher starting concentration (Figure 1B.1). *C. molischiana* and *S. cerevisiae* cdt-1 in both mono- and co-culture grew relatively rapidly, showing increases in both viable cells and optical density (Figure 1A.2,3 and B.2,3). The discrepancy between the apparent length of the growth phase in optical density for *C. molischiana* mono-cutlures and viable cells is thought to be due to the insensitivity of CFU measurements and possibly a partial loss of viable cells which still contribute to OD. During rapid growth the yeast presumably consumed all available oxygen which, in co-culture, allowed for growth of *C. phytofermentans* (Figure 1A.3 and 1B.3). This result could be expected since similar relief of oxygen inhibition has been demonstrated in fungal/bacterial and bacterial/bacterial interactions [30,31] and is generally observed in naturally occurring microbial consortia [32]. However, this is the first demonstration utilizing an anaerobic cellulolytic bacteria and cellodextrin fermenting yeast. These results confirm that yeast provide respiratory protection to the obligate anaerobe, *C. phytofermentans* as an important component of the symbiotic cooperation that would be required for growth on cellulose as the sole carbon source.

**Figure 2:**
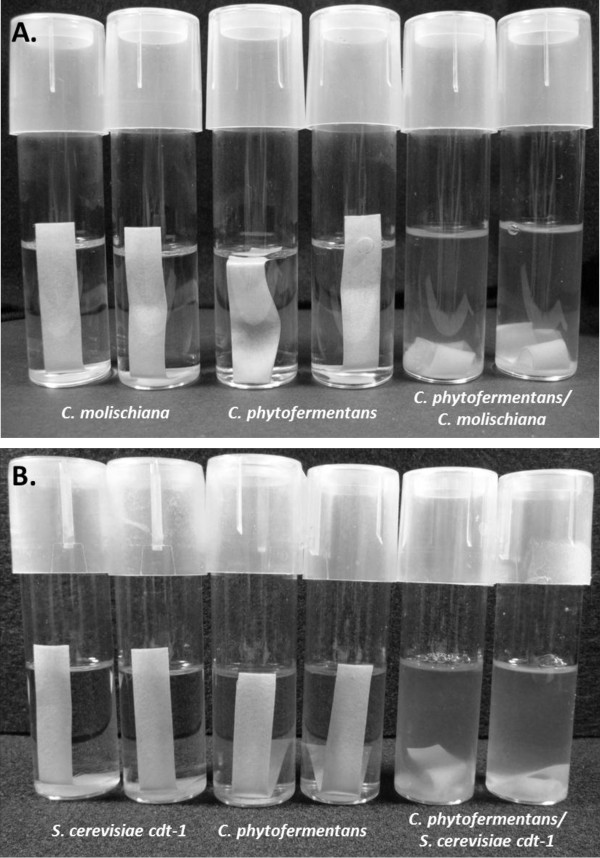
**Static mono- and co-culture hydrolysis of filter paper.** Semi-aerobic cultures in non-degassed GS2 medium with Whatman No. 1 filter paper strips as the carbon source. *C. molischiana* and *C. phytofermentans* mono- and co-cultures (**A**) and *S. cerevisiae* cdt-1 and *C. phytofermentans* mono- and co-cultures (**B**). Photographs taken after 40 days (**A**) and 15 days (**B**) static incubation at 30°C with periodic agitation. Image is representative of at least two independent experiments each with two replicates (as shown in the photographs).

This microbial interaction was expanded to include the complimentary component of the proposed symbiosis by providing carbohydrate that is released from cellulose by *C. phytofermentans*. Mono- and co-cultures were inoculated into medium containing Whatman #1 filter paper strips under semi-aerobic conditions (i.e., initially oxygenated with tubes that allowed gas transfer). After approximately 15–40 days of static incubation at 30°C, only co-cultures showed degradation of filter paper (Figure 2). Here *C. molischiana* and *S. cerevisiae* cdt-1 provide respiratory protection to *C. phytofermentans* in exchange for soluble substrate provided from cellulose via *C. phytofermentans* hydrolysis. However, subsequent experimentation at increased oxygen transfer rates showed that these soluble products were a combination cellodextrins, glucose and ethanol produced by *C. phytofermentans* (data not shown). Protective mechanisms similar to this have been observed in mixed bacterial cultures [33] which supports the use of oxygen as a robust regulatory mechanism and suggests that flexibility exists in the selection of the facultative partner organism.

### Low level oxygen transfer promotes symbiosis while maintaining ethanol production

**Figure 3:**
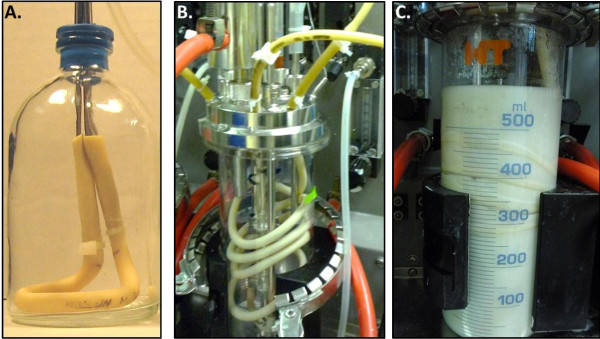
**Bioreactors designed and utilized for diffusive oxygen transfer.** Controlled oxygen transfer reactor with 10 cm neoprene tubing fixed to stainless steel tubing inserted into a sealed 100 mL serum bottle (**A**). Infors 500 mL reactor containing 100 cm neoprene tubing fixed to stainless steel outlet ports (**B**). Infors 500 mL reactor in operation with tubing loops submerged in the cellulose-containing medium (**C**).

To prevent ethanol consumption while maintaining sufficient oxygen to promote the symbiosis requires the use of controlled low level oxygen introduction. Submerged neoprene tubing with continuous internal air flow was used to transfer oxygen in two different scale reactor systems, 50 mL and 500 mL (Figure 3). Using this design, altering the submerged tubing length provides a simple means of altering the oxygen transfer rate. Assuming a constant driving force from air and a zero oxygen concentration in the culture (as determined visually by colorless resazurin) the oxygen transfer rate (OTR) through the tubing was estimated to be approximately 0.04 μmol O_2_/cm hour. With 10 cm of submerged tubing in 50 mL of medium the OTR = 8 μmol O_2_/L hour which corresponds to a culture volumetric transport rate of approximately 0.03 hour^-1^ (which is several orders of magnitude lower than typical shake flask fermentations [34]).

Using this OTR, *C. molischiana* and *S. cerevisiae* cdt-1 mono-cultures grew and fermented cellobiose with a lag phase of approximately 150–200 and 50–75 hours, respectively (Additional file 1B and F). *C. phytofermentans* mono-cultures displayed only slightly inhibited growth and ethanol production compared to no oxygen controls (Additional file 1D). Without oxygen, growth and/or cellobiose consumption were not observed in *C. molischiana* mono-cultures while gradual cellobiose consumption was observed in *S. cerevisiae* cdt-1 mono-cultures (Additional file 1A and E). In fact, even with the addition of Ergosterol and Tween 80 (two common anaerobic growth factors for yeast [35]) and replacement of cysteine with glutathione [36] we were unable to achieve growth of *C. molischiana* on cellobiose under completely anaerobic conditions. *S. cerevisiae* cdt-1, on the other hand, displayed growth under these conditions (Additional file 2). This observation agrees well with other reports that indicated only a small number of yeast are able to grow in the complete absence of oxygen [37].

As expected, in anaerobic cellobiose co-culture *C. molischiana* and *S. cerevisiae* cdt-1 were outcompeted; the viable cell counts fell below detection (< 100 CFU/mL) after about 100–200 hours (Additional file 3). On the other hand, co-cultures with oxygen transfer fermented all the cellobiose and supported the growth of both *C. phytofermentans* and *C. molischiana* or *S. cerevisiae* cdt-1 (Additional file 3). The ethanol yield of the co-culture was slightly lower than that of the yeast mono-cultures since a small amount of acetate was produced early in the fermentation. In contrast to Figure 1, *C. phytofermentans* benefited early in the culture since the media was initially anaerobic while the increase in yeast viable cell counts occurred after approximately 100–200 hours during which time oxygen was continuously diffused into the culture (Additional file 3). This OTR appeared suitable for inducing and maintaining a co-culture of *C. phytofermentans* and either *C. molischiana* or *S. cerevisiae* cdt-1 on cellobiose while allowing for high ethanol yield. Therefore, the same OTR was implemented in cellulose fermentations. In contrast to a recent co-culture CBP study which required sequential culturing conditions for complimentary function while selectively inhibiting growth [38], this approach allows simultaneous growth and fermentation of both organisms which drastically simplifies culturing techniques.

### Stable co-culture cellulose fermentation

**Figure 4:**
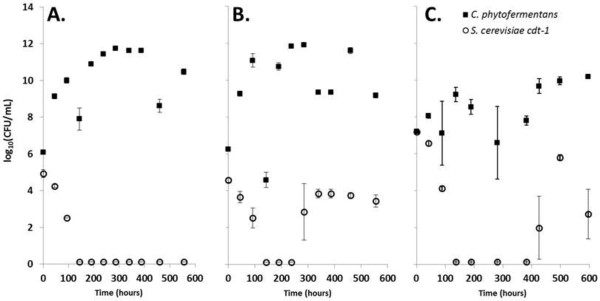
**Co-culture population dynamics with diffusive oxygen transfer.** Population dynamics for 50 mL, 25 g/L α-cellulose co-culture fermentations without added oxygen (**A**) and with added oxygen (**B**) and 500 mL, 100 g/L α-cellulose co-culture fermentation (**C**). Results in **A** and **B** are representative of at least 3 independent experiments. Results in **C** represent a single experiment. Error bars are plus and minus one standard deviation among the drops used for colony counting.

Using low oxygen transfer rates as the symbiosis mediator, co-cultures were performed with 25 g/L α-cellulose as the sole carbon source. *S. cerevisiae* cdt-1 was utilized in the remainder of the studies rather than *C. molischiana* due to its superior anaerobic growth and co-culture capabilities. *C. phytofermentans* dominated α-cellulose co-cultures during the first few hundred hours during which time yeast populations fell from about 10^6^ viable cells per mL to below detection (Figure 4A and B). Without oxygen, the yeast population remained at or below detectable limits in both co-culture (Figure 4A) and mono-culture (Additional file 4A). In co-culture fermentations with added oxygen, the yeast population recovered to a maximum of about 10^4^ – 10^5^ CFU/mL presumably due to simultaneous oxygen and carbon availability (Figure 4B). In fact, soluble cellodextrin concentrations in co-cultures with oxygen were consistently lower than those without oxygen even though cellulose hydrolysis was essentially identical (data not shown). As expected due to their inability to utilize cellulose, *S. cerevisiae* cdt-1 mono-cultures did not grow with or without added oxygen (Additional file 4A). *C. phytofermentans* growth was relatively unaffected by co-culturing (Figure 4) and oxygen introduction (Additional file 4B). This confirms that oxygen can be used to generate a symbiotic relationship between *C. phytofermentans* which provides a soluble carbon source to *S. cerevisiae* cdt-1 from cellulose in return for preventing oxygen inhibition. However, for these treatments no significant improvements in ethanol production or cellulose hydrolysis were observed in oxygen supplemented co-cultures relative to anaerobic *C. phytofermentans* mono-cultures (data not shown).

To investigate the scalability and attempt to improve performance of the co-culture, the fermentations were scaled up ten-fold (from 50 mL to 500 mL) to an Infors Sixfors bioreactor system (Figure 3C) with enhanced mixing, pH control and an α-cellulose concentration of 100 g/L. The OTR was maintained at approximately 0.4 μmol O_2_/hour by increasing the submerged tubing length by a factor of ten from 10 to 100 cm. Under these conditions, the population dynamics were essentially identical to the small scale reactors with only a slightly longer delay in *S. cerevisiae* cdt-1 re-growth (Figure 4C). Even with pH control, mixing and higher initial substrate concentration, the fermentation performance was not significantly improved compared to the small scale fermentations (data not shown). It is none-the-less encouraging that the population control mechanism based on oxygen transport rates could be readily scaled. In addition to increasing tubing length it is easy to envision that the OTR could be maintained by altering tubing thickness, internal oxygen concentration or with minimal gas sparging at a larger scale.

The long term population stability of the co-culture was determined by growing *C. phytofermentans* and *S. cerevisiae* cdt-1 in co-culture with and without oxygen for 50 days. Serum bottles with 25 mL GS2 medium and 150 g/L α-cellulose were inoculated with approximately equal cell concentration (about 10^5^ CFU/mL) of *C. phytofermentans* and *S. cerevisiae* cdt-1. At 30, 40 and 50 days the individual cell concentrations were determined (Additional file 5A and B). The co-culture with added oxygen maintained *C. phytofermentans* and *S. cerevisiae* cdt-1 concentrations of 10^5^ to 10^7^ CFU/mL at every time point whereas, without oxygen, the yeast population fell below detection prior to the 30 day sample. The presence of yeast in the oxygen positive co-culture was also apparent in that no glucose was accumulated compared to almost 3 g/L glucose accumulation in the co-culture without oxygen (Additional file 5C and D).

These results demonstrate the ability to effectively generate a stable, symbiotic co-culture of *C. phytofermentans* and *S. cerevisiae* cdt-1 via diffusion of oxygen at a rate of approximately 8 μmol O_2_/L hour. The oxygen transfer mechanism was scalable from 50 mL to 500 mL. However, the fermentations were still limited in their hydrolytic capacity meaning the co-culture would not significantly out-perform *C. phytofermentans* mono-cultures.

### Co-culture simultaneous saccharifcation and fermentation

The lack of increased performance of the co-culture relative to *C. phytofermentans* mono-culture appears to be due to an inability of *C. phytofermentans* to hydrolyze high solids loading cellulose to provide soluble substrate for the yeast partner. *C. phytofermentans* was repeatedly unable to fully utilize insoluble substrate concentrations in excess of approximately 30 g/L (unpublished observation). To explore the potential for improving the co-culture approach we sought to increase soluble sugar yield by fermenting α-cellulose with and without added endoglucanase from *Trichoderma viride* (3.2.1.4). This specific approach was taken to simulate additional hydrolysis capacity of *C. phytofermentans* while acknowledging the differences that exist between fungal and Clostridial cellulases.

**Figure 5:**
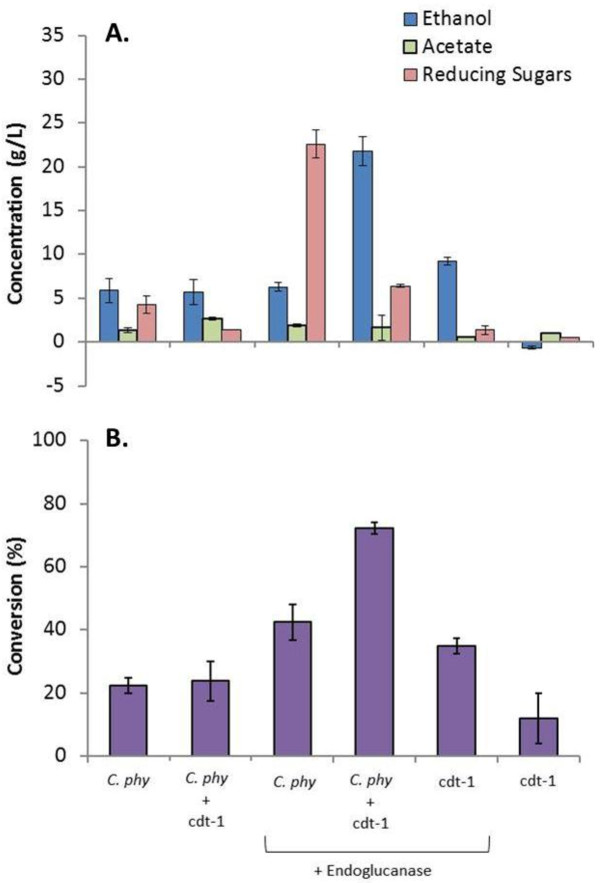
**Mono- and co-culture performance under CBP and SSF conditions.***C. phytofermentans* mono- and co-culture CBP without added enzyme and SSF with 400 mg/L endoglucanase at 30°C. Performance is shown by ethanol, acetate and accumulated reducing sugar concentrations (**A**) and cellulose conversion (**B**). Bars represent the average of two independent experiments and error bars are plus and minus one standard deviation among experiments. *C. phy* indicates *C. phytofermentans* mono-culture, cdt-1 indicates *S. cerevisiae* cdt-1 mono-culture and *C. phy* + cdt-1 indicates *C. phytofermentans*/*S. cerevisiae* cdt-1 co-culture. + Endoglucanase indicates an SSF experiment with endoglucanase added as stated in the text. *S. cerevisiae* cdt-1 mono-cultures and *C. phytofermentans/S. cerevisiae* cdt-1 culture were grown with oxygen transfer while *C. phytofermentans* mono-cultures remained completely anaerobic throughout the experiment.

Mono- and co-cultures were grown in 100 mL serum bottles with 100 g/L α-cellulose in 50 mL of ETGtGS2 medium. After about 400 hours, *C. phytofermentans* mono-cultures without oxygen hydrolyzed approximately 22% of the α-cellulose. With the addition of 0.4 g/L endoglucanase, or 3.8 IU/mL, conversion increased to about 42% but the majority of the released reducing sugars simply accumulated leaving almost 23 g/L reducing sugars in the final sample (Figure 5A). *C. phytofermentans* mono-cultures with *and* without added endoglucanase resulted in about 6 g/L ethanol suggesting a limitation in metabolic capacity of *C. phytofermentans* (Figure 5A). With oxygen, approximately 35% of the α-cellulose was hydrolyzed in *S. cerevisiae* cdt-1 mono-cultures (with enzyme) with about 1 g/L reducing sugars accumulated and 9 g/L ethanol produced. Co-cultures without added enzyme behaved essentially identical to *C. phytofermentans* mono-cultures consistent with hydrolysis-limited productivity. On the other hand, the co-culture with oxygen and endoglucanase produced more than two times more ethanol than either mono-culture with a final concentration of approximately 22 g/L and hydrolyzed twice as much cellulose with a conversion of about 72% (Figure 5). A single experiment was incubated further and with an additional 240 hours the co-culture reached almost 30 g/L ethanol and 90% cellulose conversion (Additional file 6). The *C. phytofermentans* mono-culture with and without endoglucanse failed to produce additional ethanol and the *S. cerevisiae* cdt-1 mono-cultures reached only 13 g/L after the additional 240 hours of incubation (Additional file 6).

These results demonstrate that co-culture with *C. phytofermentans* significantly enhances simultaneous saccharification and fermentation of α-cellulose with *S. cerevisiae* cdt-1. It is important to note in Figure 5 that the maximum productivity was observed for the consortium relative to the mono-culture even when exogenous cellulase was provided. This suggests there is considerable room for optimizing the productivity, and contrary to concerns of complexity, the symbiotic consortium is inherently stable. This synergistic interaction produced a final ethanol concentration that was 30% greater than the sum of the mono-culture titers (22 g/L versus 15 g/L). The improvements shown here are likely due to the combination of endoglucanse and the enzyme repertoire of *C. phytofermentans* relative to the single enzyme (endoglucanase) used in *S. cerevisiae* cdt-1 mono-cultures. The relief of feedback inhibition on *T. viride* endoglucanase by consumption of accumulated sugars and decreased pH caused by *C. phytofermentans* acetate production also likely contributed to the observed improvements. It is important, however, to note that the media conditions are optimized for co-culture growth and not necessarily mono-culture SSF productivity so changes in culturing conditions could lead to improved mono-culture performance. Independent of these subtle interpretations, these results unambiguously demonstrate the power of symbiotic microbial consortia in cellulosic ethanol production. The results also suggest that with improvements in C*. phytofermentans* hydrolytic capacity, the co-culture has the potential to reach commercially relevant ethanol concentrations from α-cellulose.

## Conclusions

**Figure 6:**
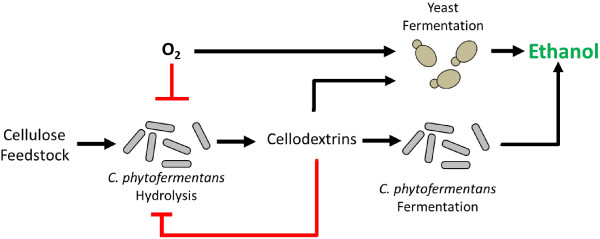
**Proposed symbiotic consortium design.***C. phytofermentans*-yeast symbiotic consortium design based on cellulose hydrolysis to soluble cellodextrins which are utilized by yeast. Both *C. phytofermentans* and yeast produce ethanol from cellodextrins. Cellodextrins feedback inhibit cellulose hydrolysis, oxygen inhibits anaerobic bacterial growth while yeast oxygen consumption relieves inhibition.

We have demonstrated the development, verification and application of a symbiotic co-culture of a cellulolytic mesophile, *C. phytofermentans*, and the cellodextrin fermenting yeast, *C. molischiana* or *S. cerevisiae* cdt-1 for direct ethanol production from α-cellulose. Controlled oxygen transfer is used to induce a symbiosis between the two organisms in which the yeast removes oxygen, protecting *C. phytofermentans*, in return for soluble carbohydrates liberated from cellulose (Figure 6). The symbiotic co-cultures were stable for almost 2 months, hydrolyzed cellulose under semi-aerobic conditions and produced more ethanol from α-cellulose via SSF than *C. phytofermentans* or *S. cerevisiae* cdt-1 mono-cultures. The addition of a moderate level of cellulase 400 mg/L (3.2.1.4 from *T. viride*) to the co-cultures in SSF experiments improved ethanol production two-fold greater than *S. cerevisiae* cdt-1 mono-culture and approximately four-fold greater than *C. phytofermentans* mono-cultures giving a final concentration of approximately 22 g ethanol/L after 400 hours. These results suggest that *C. phytofermentans* hydrolysis rates are well-matched to its natural metabolic rates and increased cellulase activity is needed to support a highly productive symbiosis. In addition, oxygen introduction as a mechanism for symbiosis establishment in microbial consortia was scaled from 50 mL to 500 mL and can be readily scaled up by conventional gas transfer at low cost making it highly applicable to industrial fermentation.

This work represents a significant advancement in establishing and controlling a microbial consortium of diverse organisms for biochemical production. In contrast to other approaches for generating symbiotic consortia that require genetic engineering, oxygen introduction is a simple, scalable technique that could be applied to organisms where genetic engineering tools are not yet available. However, both approaches have significant merit and genetic modification combined with an environmental control mechanism, like oxygen tension, could be an extremely powerful approach to consortia-mediated bioprocessing. The successful development of controllable, stable and productive engineered microbial consortia is likely to be a key advancement in many aspects of biotechnology. This approach combines complex functionality that is distributed across numerous organisms that can be independently optimized for a specific function. To gain better understanding, control and predictive capabilities, community mass and energy balance and metabolic models [39-41] may prove quite useful. Modeling combined with ecological theory could be applied to predict the fate of the populations in a consortium and the relative fitness and/or productivity of the individuals within the overall community.

Oxygen-facilitated symbiosis should be pursued as a mechanism for generating stable consortia of various other organisms including other cellulolytic bacteria and fungi as well as pentose and hexose fermenting yeasts and bacteria. In addition, other products, interactions and processes can be explored by incorporating other naturally occurring or genetically engineered organisms that have unique metabolic properties such as the production of biofuel molecules with improved energy density. As a model system, this symbiotic consortium can be used to explore the structural and functional aspects of cellulose degrading microbial communities. The work described herein used pure cellulose as a substrate but more complex lignocellulosic substrates must be investigated. The appropriate combination of organisms in a symbiotic consortium could result in an efficient consortia-mediated bioprocess which could improve the economic feasibility of lignocellulosic biofuels.

## Methods

### Media and chemical sources

GS2 medium [42] was used in all experimental cultures with the following composition per liter: 6 g Yeast Extract (BD), 2.9 g K_2_HPO_4_ (JT Baker), 2.1 g Urea (Sigma), 1.5 g KH_2_PO_4_ (JT Baker), 3 g Tri-Na Citrate H_2_O (Fischer), 2.33 g Cysteine HCl H_2_O (MP Biomedical), 1 g MOPS (Sigma), 0.1 g MgCl_2_ 6H_2_O (JT Baker), 0.0113 g CaCl_2_ Annhydrous (Fischer), and 0.000125 g FeSO_4_ 7H_2_O (Fischer) and 0.01% Resazurin (Aldrich). The salts solution (MgCl_2_ 6H_2_O, CaCl_2_ Annhydrous and FeSO_4_ 7H_2_O) and resazurin solution were made and sterilized separately and added after autoclave. The pH of the basal components was adjusted to 7 prior to sterilization. The concentration of α-Cellulose (Sigma) was as described in the text. For inoculum cultures α-Cellulose was replaced with 3 g/L Cellobiose. The GS2 medium was modified for α-cellulose co-cultures to contain 10 mg/L Ergosterol and 420 mg/L Tween 80 and 4.09 g/L Glutathione in place of 2.33 g/L Cysteine HCl H_2_O (termed ETGtGS2). Either GS2 medium without cysteine and/or glutathione or YPC (5 g/L yeast extract, 10 g/L peptone and 3 g/L cellobiose) was used for cultivation of *C. molischiana* and *S. cerevisiae* cdt-1. Half strength Sabouraud medium contained (per liter) 5 g Casien Digest (Sigma), 5 g Dextrose (EM Sciences) and 10 g Agar (BD).

### Inoculation

Initial cultures of *Clostridium phytofermentans* ISDgT were prepared from cryogenically (−80°C) frozen stocks in 7.5 mL screw cap tubes with 5 mL GS2 medium. Oxygen was removed from *C. phytofermentans* culture medium by degassing (until de-pinked) in a Coy Anaerobic Chamber with a 1.5% H_2_/98.5% N_2_ atmosphere. *C. phytofermentans* inoculum cultures were allowed to grow at 30°C to an OD_600_ of about 0.3. Cultures were then restarted by placing 100 μL of the initial culture into 4.9 mL of fresh GS2 medium. Experiments were inoculated by injecting approximately 2–3 mL of restarted cultures (OD_600_ of about 0.2-0.3) to give an initial OD_600_ of approximately 0.0125-0.025. The amount of substrates and products in the inoculum was determined by HPLC and was subtracted from the final concentrations in the cultures to determine true yields.

For cellobiose co-culture experiments and filter paper degradation experiments yeast cultures were grown in GS2 medium without cysteine and/or glutathione under aerobic conditions at 30°C until reaching an OD_600_ of at least 0.3 then restarted. The cultures were then restarted and the restarted cultures were used as inoculum to obtain an initial OD_600_ of approximately 0.0125-0.025. For the α-cellulose experiments yeast cultures were grown in YPC medium at 30°C until reaching an OD_600_ of at least 0.5, were restarted in YPC and allowed to grow until reaching an OD_600_ of approximately 1 and were then concentrated and used to inoculate with an initial OD_600_ of about 0.05.

### Experimental cultures

Fermentations were conducted in 100 mL serum vials sealed with butyl rubber stoppers and incubated at 30°C with 200 rpm agitation or in the 500 mL Infors Sixfors bioreactor system at 30°C with 300 rpm stirring. In the Sixfors bioreactor system, pH was controlled using addition of 1 N sodium hydroxide. Cultures were degassed in an anaerobic chamber to remove oxygen at the beginning of the experiment as described previously. A loop of neoprene tubing (OR = 3.175 mm, IR = 1.45 mm) was submerged in the culture medium and attached at each end to stainless steel tubing inserted through the butyl stopper. In oxygen positive cultures, air was allowed to flow through the neoprene tubing at a flow rate of about 100 L/hr using an aquarium pump. The stainless steel tubing was fashioned in a loop for oxygen negative cultures so that no oxygen could enter the culture.

Static hydrolysis experiments were conducted in 30 mL flat-bottom test tubes and incubated at 30°C with periodic agitation. Experimental media was not degassed prior to inoculation and residual carbon was not removed from the inoculum. Resazurin was not included in the media.

Simultaneous saccharification and fermentation experiments were conducted in 100 mL serum vials sealed with butyl rubber stoppers and incubated at 30°C with 200 rpm agitation as previously described. Endoglucanase (3.2.1.4 from *Trichoderma viride*) was suspended in phosphate buffered saline, degassed and injected to reach a final concentration of 400 mg/L. Cultures without endoglucanase received degassed, phosphate buffered saline only. One way valves attached to needles were inserted through the butyl stopper to release the gas produced via fermentation.

### Dry weight measurements

Cellulose dry weight samples (1 mL) were removed from reactors with a syringe and placed into pre-tared 1.7 mL eppi-tubes (VWR). The samples were centrifuged at 14,000 x g for 10 minutes and the supernatant was removed (used as HPLC sample as described in the following section). The cellulose pellet was washed with DI H_2_O and again centrifuged at 14,000 x g for 10 minutes. The supernatant was removed and the tube was placed, open, in a drying oven at 70°C. The samples were dried until reaching a constant mass and then weighed. The difference between the final and initial weight of the tube was assumed to be the dry mass of cellulose neglecting cellular mass.

### Fermentation product determination

Cellobiose, glucose, ethanol and acetate concentrations were determined from supernatant (as described previously) taken from each culture and frozen immediately at −20°C. Following the completion of the experiment the samples were thawed and filtered into 1.5 mL screw cap vials (Agilent) using 0.45 μm nylon syringe filters (Whatman). Samples were analyzed on an Agilent 1100 HPLC with a Jasco RI-1531 refractive index detector (RID) using an Aminex HPX-87H Cation exchange column (BioRad) with the appropriate guard column. Filtered (through 0.22 um filter) 0.01 M sulfuric acid was used as the mobile phase, the column temperature was set at 65°C and the RID was set at 30°C. Samples were injected at a volume of 25 uL and the operating flow rate was 0.6 mL/min. Product formation reflects correction to the initial concentration in the medium immediately following inoculation. Therefore, carryover of low concentrations of fermentation products and subsequent consumption of these products (e.g., ethanol) may lead to negative production values.

### Soluble carbohydrate determination

Soluble carbohydrates were determined using the anthrone-sulfuric acid colorimetric assay of Dreywood (Dreywood, 1946) adapted to a 96-well plate (Leyva et al. 2008). Breifly, 50 μL of appropriately diluted sample was mixed with 150 μL anthrone reagent (2 g/L anthrone in 98% sulfuric acid) in a polypropylene 96-well plate and covered with a nylon adhesive cover. The 96-well plates were incubated at 4°C for 10 minutes, 100°C for 20 minutes and room temperature for 20 minutes. The nylon cover was then removed and the plate was read in a Spectramax 384 Plus spectrophotometer at 620 nm. The soluble carbohydrate concentration was determined using a known concentration standard curve that was run with each 96-well plate used.

### Colony forming unit determination

*C. phytofermentans* and yeast populations were determined by performing serial dilutions of experimental samples in 96-well plates and subsequent drop plating of each dilution on the appropriate medium. *C. phytofermentans* was plated on GS2 medium with 6 g/L lactose and was incubated at 30°C in an anaerobic chamber. *C. molischiana* and *S. cerevisiae* cdt-1 were plated on ½ Sabouraud medium and incubated at 30°C under aerobic conditions. Co-culture samples were plated on both types of plates and incubated under their respective conditions. Colonies were counted on dilutions that contained approximately 3 to 30 colonies per drop. Due to the non-soluble nature of cellulose which may act to trap, attach to or reject cells, colony counts often displayed significant variability but trends were found to be consistent across experiments.

## Abbreviations

AFEX: Ammonia fiber expansion; OD600: Optical density at 600 nm; CBP: Consolidated bioprocessing; OTR: Oxygen transfer rate; HPLC: High performance liquid chromatography; CFU: Colony forming unit; IU: International units; SSF: Simultaneous saccharificaiton and fermentation

## Competing interests

The authors declare that they have no competing interests.

## Author contributions

TZ and WC conceived and designed the study. TZ carried out the majority of the experiments and drafted the manuscript. SX conducted the *C. phytofermentans/C. molischiana* static hydrolysis experiments and analysis as well as participated in media design. TZ and WC edited the draft. All authors read and approved the final manuscript.

## Supplementary Material

Additional file 1 **Mono-culture growth and cellobiose fermentation performance with and without diffusive oxygen transfer.** Representative OD (top figures) and consumption/production profiles (bottom figures) for *C. molischiana* mono-cultures without oxygen (A), *C. molischiana* mono-cultures with oxygen (B), *C. phytofermentans* mono-cultures without oxygen (C),*C. phytofermentans* mono-cultures with oxygen (D), *S. cerevisiae* cdt-1 without oxygen (E) and *S. cerevisiae* cdt-1 with oxygen (F). Cellobiose (filled diamonds) and ethanol (open circles) are in grams per liter. Other products (e.g., glucose and acetate) are not shown for simplicity. Data is representative of at least two independent experiments. Click here for file

Additional file 2 **Growth of *S. cerevisiae *cdt-1 and *C. molischiana *in improved media under anaerobic conditions.** Growth of *S. cerevisiae* cdt-1 (squares) and *C. molischiana* (circles) in GS2 medium containing ergosterol, Tween 80 and glutathione in place of cysteine. Closed and open symbols represent two different experimental trials where each symbol is the average of two replicates. Error bars denote plus and minus one standard deviation between the replicates. Note that aerobic controls of both organisms grew well under these conditions. Click here for file

Additional file 3 **Co-culture growth and cellobiose fermentation performance with and without diffusive oxygen transfer.** Representative CFU/mL (top figures) and consumption/production profiles (bottom figures) for *C. phytofermentans*/*C. molischiana* co-cultures (A) and *C. phytofermentans/S. cerevisiae* cdt-1 co-cultures (B) Cellobiose (filled diamonds) and ethanol (open circles) are in grams per liter. Data is representative of at least two independent experiments. Click here for file

Additional file 4 **Mono-culture population dynamics with and without diffusive oxygen transfer.** Population dynamics for 50 mL, 25 g/L α-cellulose *S. cerevisiae* cdt-1 mono-culture fermentations (A) and *C. phytofermentans* mono-culture fermentations (B). Results are representative of at least 3 independent experiments and error bars are plus and minus one standard deviation among the drops used for colony counting. Click here for file

Additional file 5 **Co-culture populations and soluble sugar accumulation when grown on α-cellulose with and without oxygen.** Viable cell counts at 30, 40 and 50 days for *C. phytofermentans*/*S. cerevisiae* cdt-1 co-cultures without oxygen (A) and with oxygen (B). Accumulated soluble sugars (including glucose) and glucose concentration at 30, 40 and 50 days for *C. phytofermentans*/*S. cerevisiae* cdt-1 co-cultures without oxygen (C) and with oxygen (D). Bars are the average of two replicates and error bars represent plus and minus one standard deviation. Click here for file

Additional file 6 **Mono- and co-culture performance under CBP and SSF conditions after 640 hours.***C. phytofermentans* mono- and co-culture CBP without added enzyme and SSF with 400 mg/L endoglucanase at 30°C. Performance is shown by ethanol and acetate concentrations (A) and cellulose conversion (B). Bars represent the average of two replicates in a single experiment and error bars are plus and minus one standard deviation among replicates. *C. phy* indicates *C. phytofermentans* mono-culture, cdt-1 indicates *S. cerevisiae* cdt-1 mono-culture and *C. phy* + cdt-1 indicates *C. phytofermentans*/*S. cerevisiae* cdt-1 co-culture. + Endoglucanase indicates an SSF experiment with endoglucanase added as stated in the text. *S. cerevisiae* cdt-1 mono-cultures and *C. phytofermentans/S. cerevisiae* cdt-1 culture were grown with oxygen transfer while *C. phytofermentans* mono-cultures remained completely anaerobic throughout the experiment. Click here for file
