# Supplementary figures and images for: Consortia-mediated bioprocessing of cellulose to ethanol with a symbiotic Clostridium phytofermentans/yeast co-culture
Source: Biotechnol Biofuels. 2013 Apr 29;6:59. doi: 10.1186/1754-6834-6-59 (PMC3653780; doi:10.1186/1754-6834-6-59)

Additional file 1

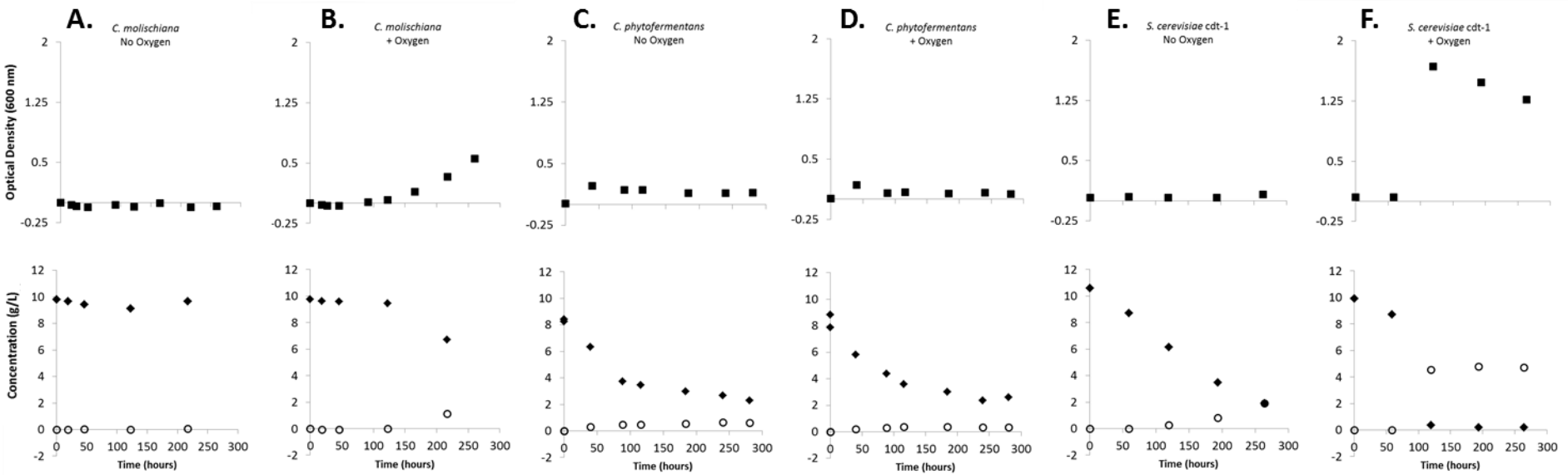

Supplement: Additional file 1 — Mono-culture growth and cellobiose fermentation performance with and without diffusive oxygen transfer. Representative OD (top figures) and consumption/production profiles (bottom figures) for C. molischiana mono-cultures without oxygen (A), C. molischiana mono-cultures with oxygen (B), C. phytofermentans mono-cultures without oxygen (C),C. phytofermentans mono-cultures with oxygen (D), S. cerevisiae cdt-1 without oxygen (E) and S. cerevisiae cdt-1 with oxygen (F). Cellobiose (filled diamonds) and ethanol (open circles) are in grams per liter. Other products (e.g., glucose and acetate) are not shown for simplicity. Data is representative of at least two independent experiments. [file 1754-6834-6-59-S1.pdf]

Additional file 2

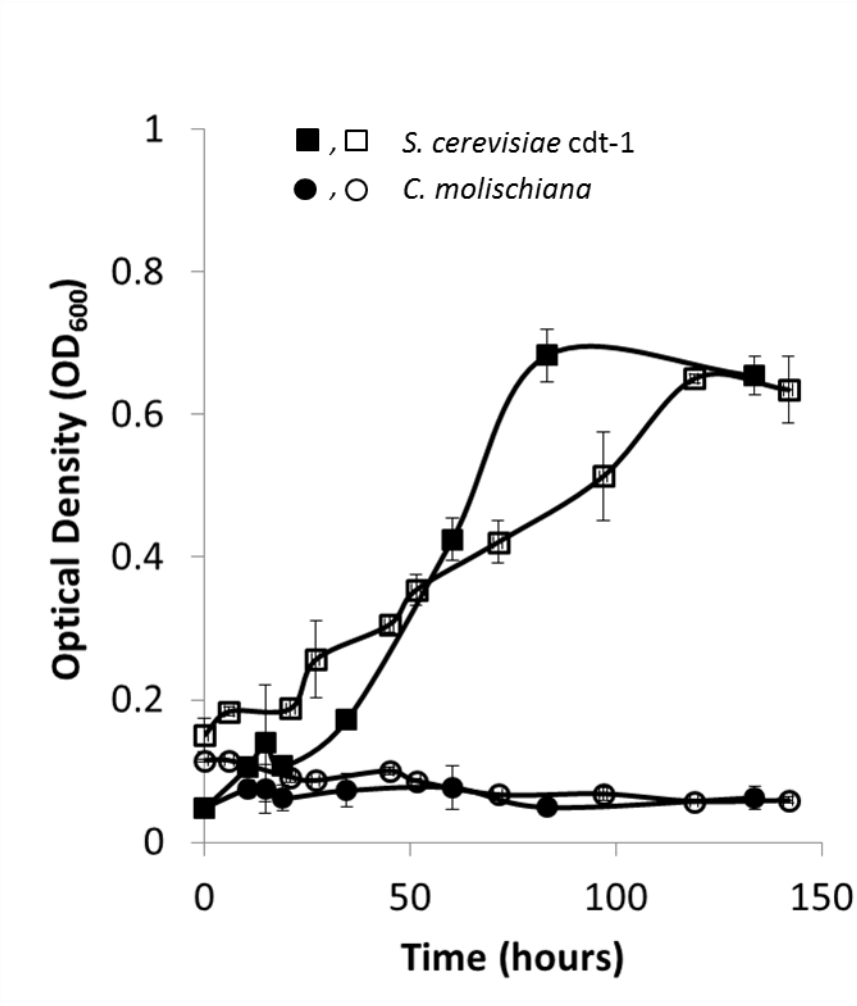

Supplement: Additional file 2 — Growth of S. cerevisiae cdt-1 and C. molischiana in improved media under anaerobic conditions. Growth of S. cerevisiae cdt-1 (squares) and C. molischiana (circles) in GS2 medium containing ergosterol, Tween 80 and glutathione in place of cysteine. Closed and open symbols represent two different experimental trials where each symbol is the average of two replicates. Error bars denote plus and minus one standard deviation between the replicates. Note that aerobic controls of both organisms grew well under these conditions. [file 1754-6834-6-59-S2.pdf]

Additional file 3

**A.** *C. phytofermentans*/*C. molischiana*

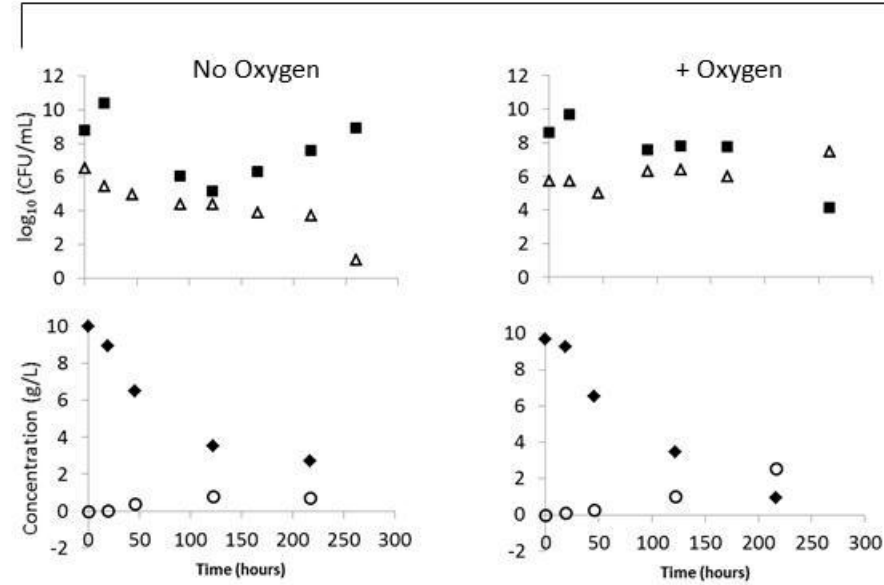

**B.** *C. phytofermentans*/*S. cerevisiae* cdt-1

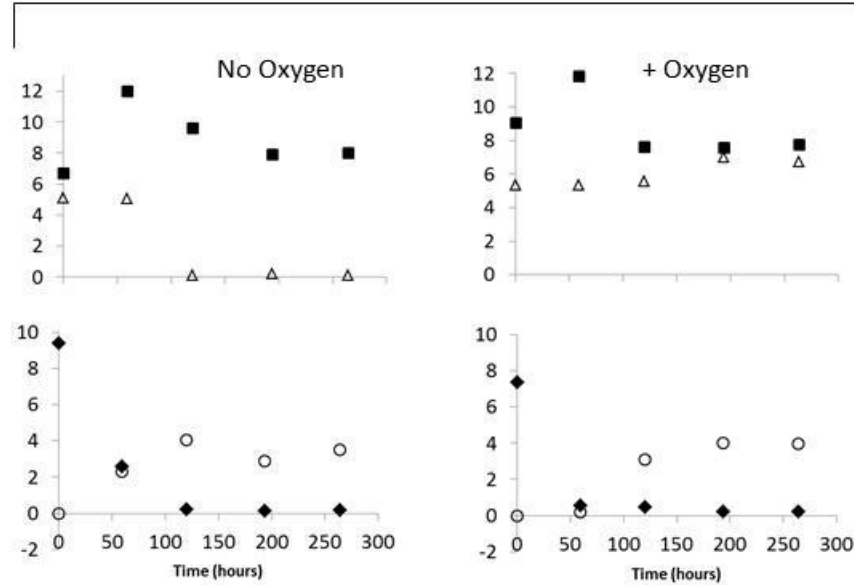

Supplement: Additional file 3 — Co-culture growth and cellobiose fermentation performance with and without diffusive oxygen transfer. Representative CFU/mL (top figures) and consumption/production profiles (bottom figures) for C. phytofermentans/C. molischiana co-cultures (A) and C. phytofermentans/S. cerevisiae cdt-1 co-cultures (B) Cellobiose (filled diamonds) and ethanol (open circles) are in grams per liter. Data is representative of at least two independent experiments. [file 1754-6834-6-59-S3.pdf]

## Additional file 4

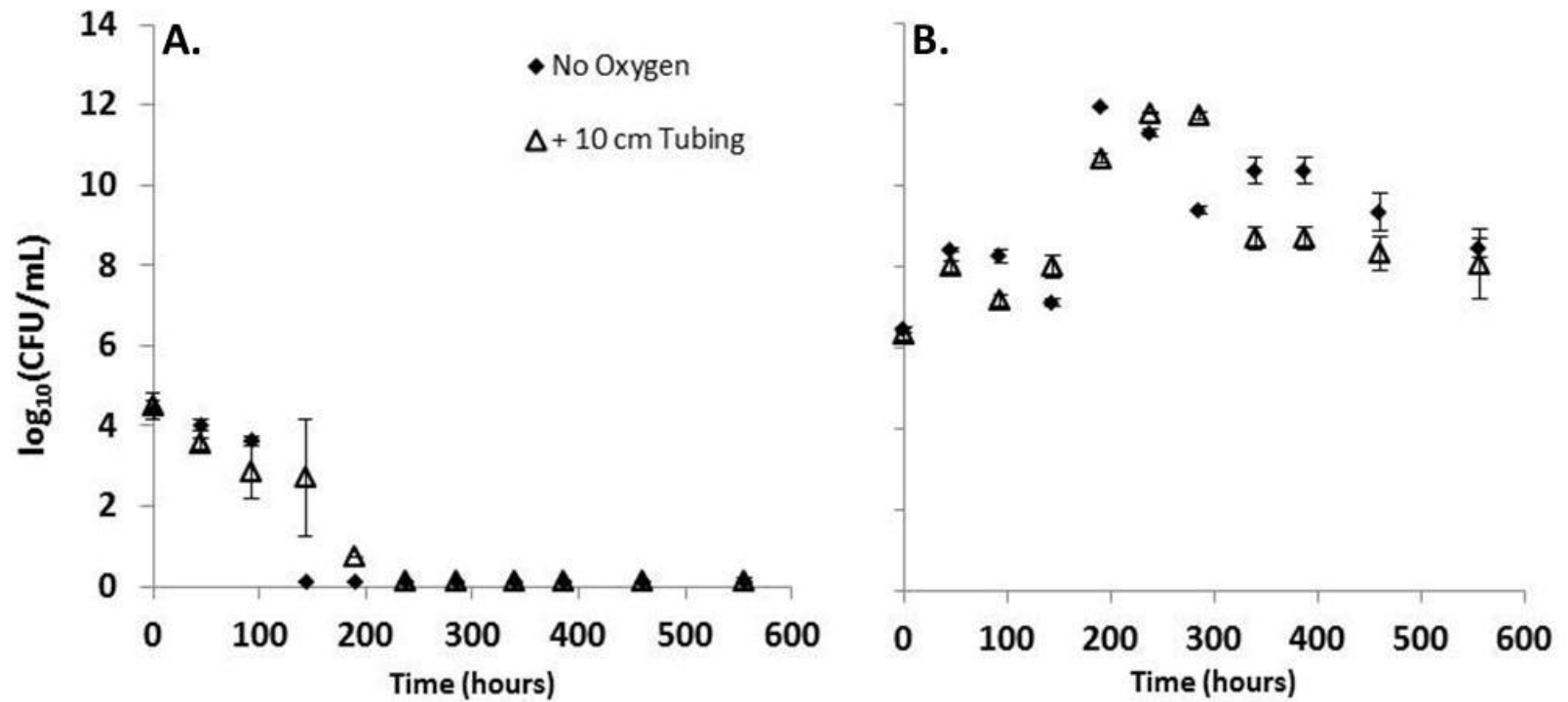

Supplement: Additional file 4 — Mono-culture population dynamics with and without diffusive oxygen transfer. Population dynamics for 50 mL, 25 g/L α-cellulose S. cerevisiae cdt-1 mono-culture fermentations (A) and C. phytofermentans mono-culture fermentations (B). Results are representative of at least 3 independent experiments and error bars are plus and minus one standard deviation among the drops used for colony counting. [file 1754-6834-6-59-S4.pdf]

## Additional file 5

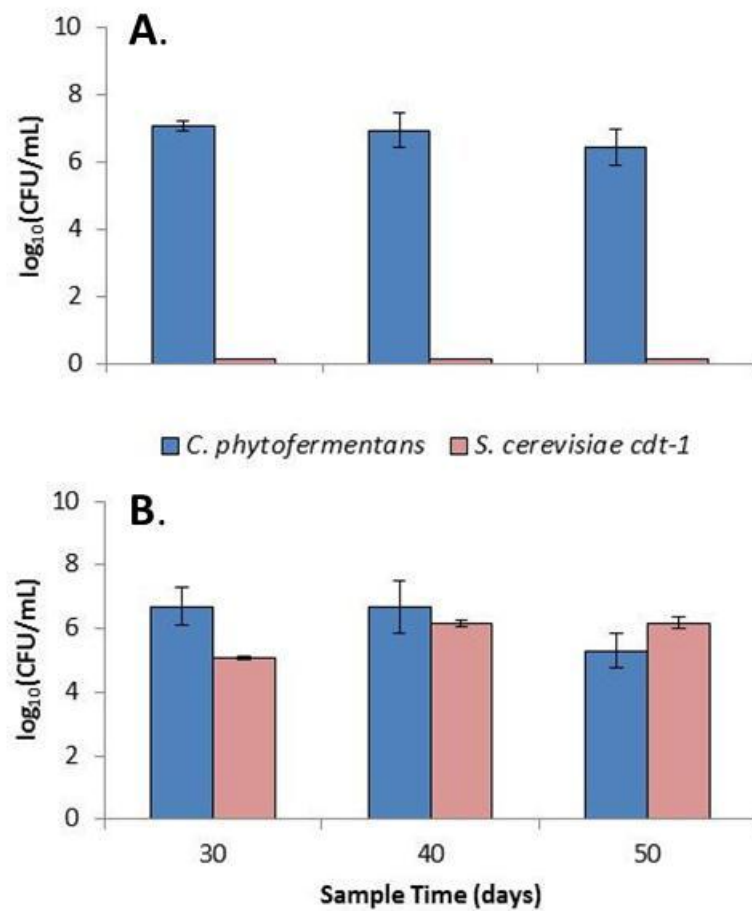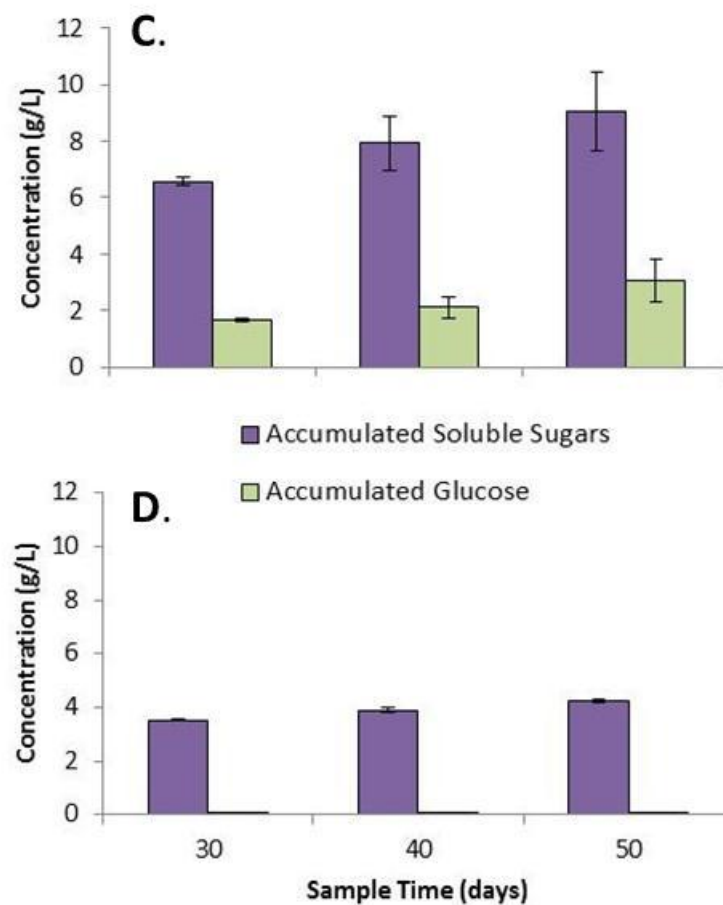

Supplement: Additional file 5 — Co-culture populations and soluble sugar accumulation when grown on α-cellulose with and without oxygen. Viable cell counts at 30, 40 and 50 days for C. phytofermentans/S. cerevisiae cdt-1 co-cultures without oxygen (A) and with oxygen (B). Accumulated soluble sugars (including glucose) and glucose concentration at 30, 40 and 50 days for C. phytofermentans/S. cerevisiae cdt-1 co-cultures without oxygen (C) and with oxygen (D). Bars are the average of two replicates and error bars represent plus and minus one standard deviation. [file 1754-6834-6-59-S5.pdf]

## Additional file 6

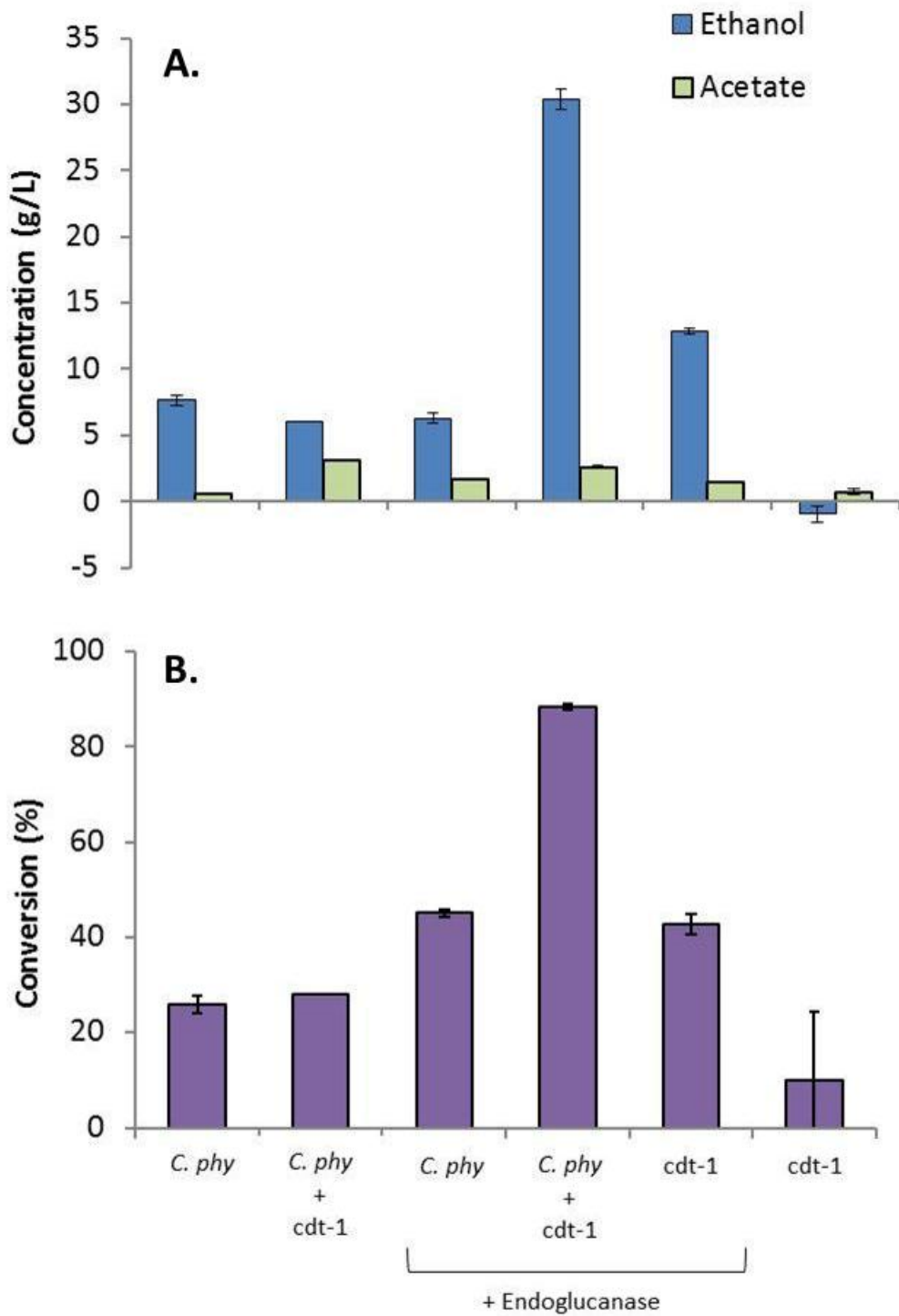

Supplement: Additional file 6 — Mono- and co-culture performance under CBP and SSF conditions after 640 hours.C. phytofermentans mono- and co-culture CBP without added enzyme and SSF with 400 mg/L endoglucanase at 30°C. Performance is shown by ethanol and acetate concentrations (A) and cellulose conversion (B). Bars represent the average of two replicates in a single experiment and error bars are plus and minus one standard deviation among replicates. C. phy indicates C. phytofermentans mono-culture, cdt-1 indicates S. cerevisiae cdt-1 mono-culture and C. phy + cdt-1 indicates C. phytofermentans/S. cerevisiae cdt-1 co-culture. + Endoglucanase indicates an SSF experiment with endoglucanase added as stated in the text. S. cerevisiae cdt-1 mono-cultures and C. phytofermentans/S. cerevisiae cdt-1 culture were grown with oxygen transfer while C. phytofermentans mono-cultures remained completely anaerobic throughout the experiment. [file 1754-6834-6-59-S6.pdf]
